# Delayed-Onset Severe Statin-Induced Rhabdomyolysis Due to Atorvastatin-Ticagrelor Interaction in a Patient With Acute Coronary Syndrome: A Case Report

**DOI:** 10.7759/cureus.111130

**Published:** 2026-06-19

**Authors:** Arwa Fareah Ansar, S M Shariar Islam, Ishma Aijazi, Jamila Bin Adi

**Affiliations:** 1 Department of Internal Medicine, Mohammed Bin Rashid University of Medicine and Health Sciences, Dubai Health, Dubai, ARE; 2 Department of Internal Medicine, Dubai Hospital, Dubai Health, Dubai, ARE; 3 Department of Internal Medicine, Rashid Hospital, Dubai Health, Dubai, ARE

**Keywords:** acute coronary syndrome, acute kidney injury, atorvastatin, drug-drug interaction, statin induced rhabdomyolysis, ticagrelor

## Abstract

Rhabdomyolysis is a rare but potentially life-threatening complication of statin therapy, typically occurring within days to weeks (mean onset approximately nine days) after initiation or dose escalation. Delayed-onset presentations after a period of uneventful use are uncommon and may delay diagnosis.

We report a 62-year-old man with ischemic heart disease and chronic kidney disease (CKD stage 3a) who developed a five-day history of bilateral lower limb pain, difficulty ambulating, and dark-colored urine. During a routine post-percutaneous coronary intervention (PCI) follow-up visit, subtle lower limb weakness was incidentally noted by his cardiologist, prompting immediate referral to the emergency department for further evaluation. He had been initiated on high-intensity atorvastatin (80 mg daily) two months following PCI. There was no history of trauma, immobilization, or recent strenuous physical activity. Laboratory investigations revealed a creatine kinase level peaking at 245,550 U/L (normal <190 U/L) and a serum creatinine of 5.22 mg/dL (normal 0.7-1.2 mg/dL, patient baseline 1.6 mg/dL), consistent with severe rhabdomyolysis complicated by acute kidney injury. Urine dipstick testing was positive for blood, while microscopic examination showed few red blood cells, consistent with myoglobinuria rather than hematuria; the autoimmune myositis workup was negative. Atorvastatin was discontinued on admission, and the patient was managed with aggressive intravenous fluid resuscitation. Renal function initially worsened but subsequently recovered without the need for renal replacement therapy.

This case highlights that statin-associated muscle toxicity can occur after a period of prior tolerance. It may present in a delayed fashion, particularly when a CYP3A4 inhibitor such as ticagrelor is co-administered. Early recognition and prompt withdrawal of the offending agent are essential to prevent severe complications. In patients requiring ongoing lipid-lowering therapy, consideration should be given to alternative agents with minimal CYP3A4 metabolism and lower potential for clinically significant drug interactions.

## Introduction

Statins are the cornerstone medications for reducing atherosclerotic risk and preventing cardiovascular disease [1]. Although they are generally well tolerated, statin-associated muscle toxicity remains a recognized adverse effect encompassing a spectrum of symptoms, ranging from mild myalgia, muscle tenderness, and cramps to proximal muscle weakness and, in its most severe form, rhabdomyolysis [2]. Rhabdomyolysis is characterized by skeletal muscle breakdown leading to the release of intracellular contents, including myoglobin, creatine kinase (CK), and electrolytes into the systemic circulation, resulting in myoglobinuria and potential acute kidney injury. It is a rare but serious complication of statin therapy, with an estimated incidence of less than 0.01% in clinical practice, typically at higher doses [3].

Patients at increased risk for statin-associated myopathy symptoms include those with advanced age, low body mass index, female sex, diabetes mellitus, hypothyroidism, chronic liver disease, chronic kidney disease (CKD), alcohol use, and vigorous exercise. Additional contributors include high-dose statin therapy, underlying neuromuscular disorders, concomitant medications, and genetic variants affecting statin metabolism [4].

Atorvastatin, particularly at high-intensity doses (80 mg daily), is a potent lipid-lowering agent frequently prescribed following percutaneous coronary intervention (PCI) for secondary cardiovascular prevention and reduction of adverse events. Atorvastatin is primarily metabolized by CYP3A4; co-administration with CYP3A4 inhibitors can increase atorvastatin plasma exposure and the risk of myotoxicity. While statin-induced rhabdomyolysis typically occurs shortly after treatment initiation or dose escalation (mean time of onset is approximately nine days), delayed presentations are uncommon and may present a diagnostic challenge [5].

We report a case of delayed-onset severe rhabdomyolysis in a 62-year-old man two months after initiation of maximum-dose atorvastatin therapy following coronary artery stenting.

## Case presentation

A 62-year-old man with a history of CKD (stage 3a, baseline serum creatinine 1.6 mg/dL with estimated glomerular filtration rate (eGFR) 47 mL/min/1.73 m², of unclear etiology), hypertension, and ischemic heart disease presented to the emergency department with a five-day history of bilateral lower limb pain, difficulty ambulating, and dark-colored urine. Two months prior, he had undergone PCI with coronary stent placement for non-ST-elevation myocardial infarction. He was subsequently initiated on atorvastatin 80 mg once daily, aspirin 75 mg once daily, ticagrelor 90 mg twice daily, bisoprolol 2.5 mg once daily, spironolactone (dose not documented in available records), and pantoprazole 40 mg once daily.

The patient denied preceding trauma, excessive physical exertion, prolonged immobilization, fever, seizures, alcohol binges, or illicit drug use. He had no history suggestive of inflammatory myopathy or thyroid disease. There were no recent infectious symptoms or sick contacts.

During a routine post-PCI follow-up visit with his cardiologist, approximately two months after statin initiation, subtle proximal lower limb weakness was incidentally noted. Given the clinical concern, he was immediately referred to the emergency department for further evaluation. On presentation, he was fully alert, afebrile, and hemodynamically stable. Examination revealed proximal muscle weakness with associated muscle tenderness. He reported that the lower limb pain and functional decline had progressively developed over the preceding five days. There was no rash, jaundice, or neurological deficit.

**Figure 1:**
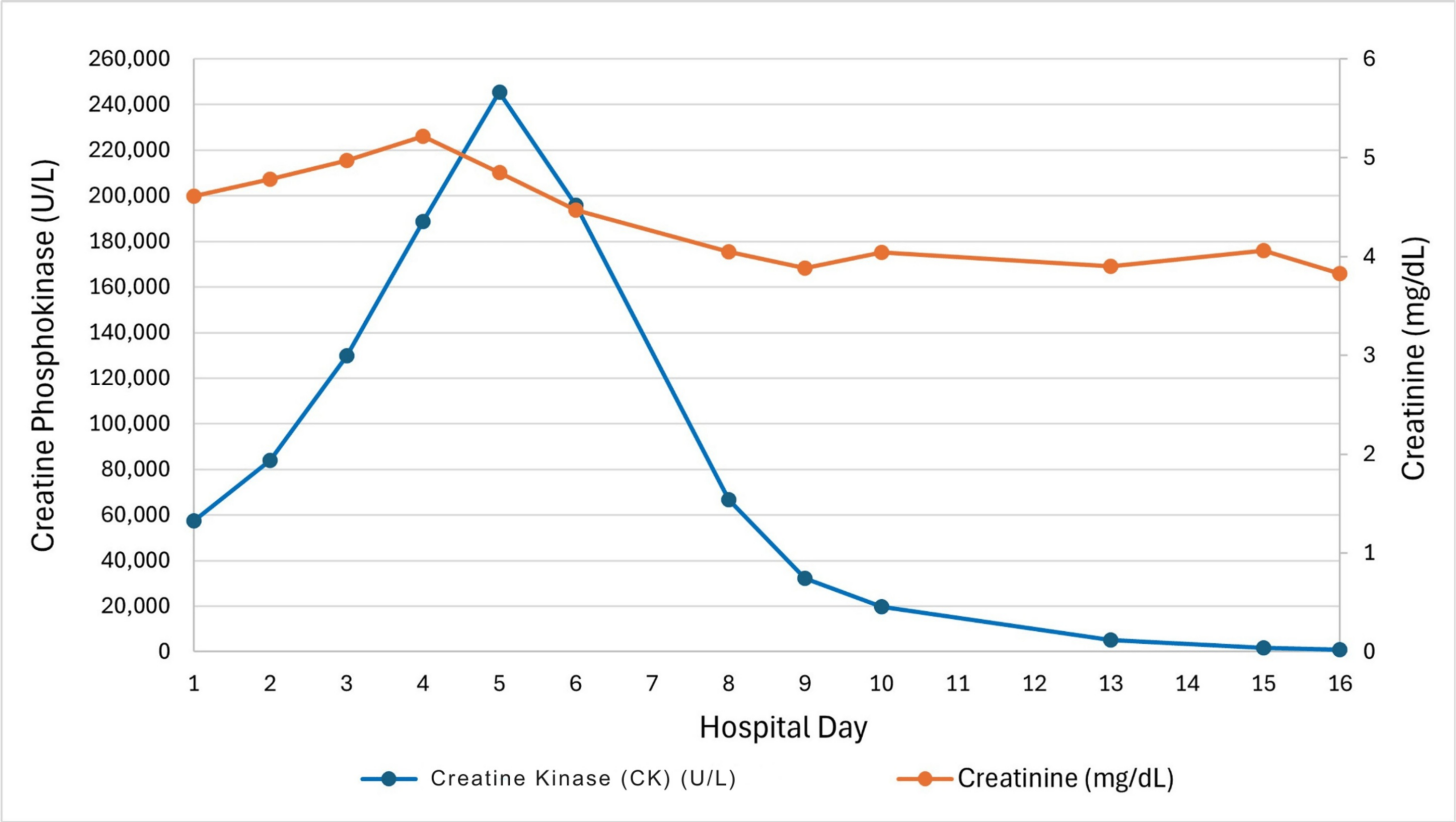
Serial trends in serum CK and creatinine levels during hospitalization Serial trends of serum CK and serum creatinine during hospitalization were plotted against hospital day. Atorvastatin was discontinued on admission, and intravenous hydration was initiated. CK levels peaked on hospital day 5 at 245,550 U/L, then declined progressively. Serum creatinine peaked at 5.22 mg/dL and subsequently improved with supportive management. CK: creatine kinase

Blood tests confirmed severe rhabdomyolysis with acute kidney injury (Table 1). CK on admission was 57,331 U/L (normal <190 U/L) and peaked at approximately 245,550 U/L over the following days (Figure 1). Serum creatinine increased from 4.61 mg/dL on admission to a peak of 5.22 mg/dL on hospital day 4 (Figure 1). Urine was dark, and the dipstick showed 3+ blood with relatively few red cells on microscopy, a pattern consistent with myoglobinuria rather than hematuria, and urine myoglobin was confirmed at 530 µg/L (normal <50 µg/L) (Table 1). Liver function tests revealed elevated transaminases, most likely reflecting muscle enzyme spillover rather than hepatocellular injury. Serum bicarbonate on admission from serum chemistry was 13.7 mmol/L, consistent with significant metabolic acidosis (venous blood gas: pH 7.318, pCO₂ 30.5 mmHg, HCO₃⁻ 15.6 mmol/L) (Table 1). Serum potassium was within the normal range on admission (4.2 mmol/L) (Table 1) despite acute kidney injury and metabolic acidosis. A mild rise in serum potassium was observed during hospitalization, peaking at 4.9 mmol/L on hospital day 4, after which sodium zirconium cyclosilicate was administered and a low-potassium diet was initiated. Metabolic acidosis was managed with oral sodium bicarbonate (1300 mg three times daily). Electrolyte levels subsequently improved with supportive management and close monitoring, with a progressive decline in serum potassium toward discharge. A progressive drop in hemoglobin from 12.2 g/dL to 10.2 g/dL, and hematocrit from 36.8% to 30.2%, with no evidence of bleeding, was observed over the following days. Given the absence of hemodynamic compromise, the patient was managed conservatively with oral iron supplementation. Hepatitis B serology demonstrated an isolated reactive hepatitis B core antibody (HBsAg non-reactive, anti-HBs non-reactive), without clinical or biochemical evidence of active hepatitis, consistent with either a prior resolved infection or false positivity.

**Table 1:** Initial laboratory and imaging investigations demonstrating severe rhabdomyolysis and acute kidney injury on admission Admission laboratory values are shown. Peak CK during hospitalization was 245,550 U/L (Figure 1). WBC: white blood cell count, RBC: red blood cell count, HPF: high-power field, MCV: mean corpuscular volume, eGFR: estimated glomerular filtration rate, ALT: alanine aminotransferase, AST: aspartate aminotransferase, TSH: thyroid-stimulating hormone, Free T4: free thyroxine, CK: creatine kinase

Lab test	Result	Reference range
Full blood count		
WBC (×10^3^/µL)	8.2	3.6-11
RBC (×10^6^/µL)	4.24	4.5-5.5
Hemoglobin (g/dL)	12.2	13-17
Hematocrit (%)	36.8	40-50
MCV (fL)	86.8	77-95
Platelet (×10^3^/µL)	146	150-410
Renal function and electrolytes		
Creatinine (mg/dL)	4.61	0.7-1.2
eGFR (mL/min/1.73m^2^)	13.6	>60
Urea (mg/dL)	169	12-40
Sodium (mmol/L)	135	136-145
Potassium (mmol/L)	4.2	3.4-4.5
Chloride (mmol/L)	105	98-108
Bicarbonate (mmol/L)	13.7	20-28
Anion Gap (mmol/L)	16	6-14
Venous blood gas		
pH	7.318	7.35-7.45
pCO_2 _(mmHg)	30.5	40-50
HCO_3 _(mmol/L)	15.6	21-28
Base (ECF)(-) (mmol/L)	10.5	
Liver function tests		
ALT (U/L)	569	0-41
AST (U/L)	1592	0-40
Total bilirubin (mg/dL)	0.76	0-1.2
Total protein (g/dL)	7.2	6.6-8.7
Albumin (g/dL)	3.9	4.4-5.1
Inflammatory markers		
C-reactive protein (mg/L)	9.6	<5
Procalcitonin (ng/mL)	0.3	<0.05
Muscle injury marker		
CPK (U/L)	57,331	<190
Urinalysis		
Urine color	Red	Yellow
Urine clarity	Hazy	Clear
Urine protein	1+	Negative
Blood by strip	3+	Negative
WBC/HPF	5 - 10	0-5
RBC/ HPF	10 -15	0-2
Urine myoglobin (µg/L)	530	<50
Thyroid function test		
Free T4 (pmol/L)	12.9	12-22
TSH (µIU/mL)	3.56	0.27-4.2
Cultures		
Blood culture	Negative	Negative
Urine culture	Negative	Negative
Imaging		
Non-contrast CT brain	Chronic microvascular ischemic changes; no acute intracranial pathology	
CT KUB	No obstructive renal or ureteric calculi	
Abdominal ultrasound	Mild fatty liver; bilateral renal parenchymal disease	

The patient had detailed investigations done to rule out other causes of rhabdomyolysis (Table 2). Since the remaining workup was negative, the close temporal association between initiation of high-dose atorvastatin and symptom onset strongly supported statin-associated rhabdomyolysis, likely potentiated by a drug-drug interaction with ticagrelor.

**Table 2:** Extended myositis and autoimmune serological workup to exclude inflammatory myopathy An extended autoimmune and myositis-specific antibody panel was performed to evaluate for inflammatory myopathy as a potential cause of rhabdomyolysis. All antibodies were negative unless otherwise indicated. Mi-2: Mi-2 nuclear helicase antibody, TIF1: transcription intermediary factor 1 antibody, MDA5: melanoma differentiation-associated protein 5 antibody, NXP2: nuclear matrix protein 2 antibody, SAE1: small ubiquitin-like modifier activating enzyme 1 antibody, Ku (Mi-2): Ku antigen antibody, PM-Scl: polymyositis/scleroderma antibody, Jo-1: histidyl-tRNA synthetase antibody, SRP: signal recognition particle antibody, PL-7: threonyl-tRNA synthetase antibody, PL-12: alanyl-tRNA synthetase antibody, EJ: glycyl-tRNA synthetase antibody, OJ: isoleucyl-tRNA synthetase antibody, Ro-52: Ro52/TRIM21 antibody, ANA: antinuclear antibody, ENA: extractable nuclear antigen, RNP-Sm: ribonucleoprotein/Smith antibody, Sm: Smith antibody, SS-A (Ro): Sjögren syndrome antigen A antibody, SS-B (La): Sjögren syndrome antigen B antibody, Scl-70: topoisomerase I antibody

Test/antibody	Result
Mi-2 alpha abs.	Negative
Mi-2 beta abs.	Negative
TIF1 abs.	Negative
MDA5 abs.	Negative
NXP2 abs.	Negative
SAE1 abs.	Negative
Mi50 (Ku) abs.	Negative
PM-Scl 100 abs.	Negative
PM-Scl 75 abs.	Negative
JO-1 abs.	Negative
SRP abs.	Negative
PL-7 abs.	Negative
PL-12 abs.	Negative
EJ abs.	Negative
OJ abs.	Negative
Ro-52 abs.	Negative
ENA RNP-SM	Negative
ENA SM	Negative
ENA SS-A (RO)	Negative
ENA SS-B (LA)	Negative
ENA scleroderma SCL-70	Negative
ENA JO-1	Negative
ENA centromeres	Negative
ANA	Negative

Atorvastatin was discontinued on the day of admission, and aggressive intravenous fluid replacement was initiated, targeting around four liters daily. Early referral to nephrology was made.

Despite the severity of the biochemical abnormalities, the patient maintained adequate urine output throughout hospitalization. The patient did not develop the anticipated complications, including severe hyperkalemia, fluid overload, or a metabolic derangement that would have necessitated initiation of dialysis. Renal function and CK levels improved progressively during hospitalization, with marked reductions in both parameters by discharge (creatinine 3.83 mg/dL; CK 969 U/L) from peak values of 5.22 mg/dL and 245,550 U/L, respectively (Figure 1). He was discharged in stable condition, with follow-up arranged with both nephrology and cardiology.

## Discussion

Statin-induced rhabdomyolysis is a rare but potentially life-threatening complication of lipid-lowering therapy [2,3]. Although most cases occur shortly after treatment initiation or dose escalation, delayed presentations occurring weeks to months later have been reported and may create a diagnostic dilemma [5]. This case demonstrates such an atypical presentation, with severe rhabdomyolysis developing two months after initiation of high-dose atorvastatin following PCI, in the absence of trauma, strenuous exercise, infection, or other identifiable precipitating factors.

The most plausible mechanism is a pharmacokinetic interaction between atorvastatin and ticagrelor. Atorvastatin is primarily metabolized via the CYP3A4 pathway, which is inhibited by ticagrelor. Pharmacokinetic data suggest that ticagrelor can increase atorvastatin plasma exposure by approximately 36% [6]. In the setting of high-dose atorvastatin therapy (80 mg daily), this increase may have contributed to elevated plasma concentrations and subsequent skeletal muscle toxicity. The presence of CKD (stage 3a, baseline serum creatinine 1.6 mg/dL with eGFR of 47 mL/min/1.73 m²) likely further increased susceptibility, as impaired renal reserve and uremic toxins are recognized risk factors for statin-associated muscle injury [2,4]. Together, these factors likely created a synergistic effect leading to severe myotoxicity.

A systematic evaluation excluded alternative etiologies. Thyroid function was normal, and an extensive myositis and autoimmune serological panel was negative, making inflammatory or endocrine myopathy unlikely. The temporal relationship between statin initiation and symptom onset, combined with clinical improvement following drug discontinuation, strongly supports a diagnosis of statin-associated rhabdomyolysis.

Management consisted of immediate discontinuation of atorvastatin and early referral to nephrology for aggressive intravenous isotonic saline infusion, initiated at approximately 150 mL/hour and subsequently titrated based on clinical response and urine output. Oral intake was encouraged, and total daily fluid intake was maintained at approximately 4 liters during the early phase of hospitalization, with higher urine outputs observed in response. Sodium bicarbonate therapy was also administered to address metabolic acidosis and urinary alkalinization in the setting of myoglobinuria [7]. Although renal function initially worsened, with creatinine peaking at 5.22 mg/dL, urine output remained adequate, and there were no absolute indications for renal replacement therapy. Conservative management was therefore continued, resulting in progressive improvement in both renal function and CK levels. This case highlights that decisions regarding dialysis in rhabdomyolysis should be guided by clinical parameters rather than biochemical severity alone [7].

A gradual decline in hemoglobin from 12.4 to 10.2 g/dL was also observed in the absence of overt bleeding. This finding likely reflects heme pigment-mediated iron sequestration and functional iron deficiency secondary to myoglobin breakdown, a recognized but underappreciated phenomenon in severe rhabdomyolysis [5].

Given the association and suspected drug interaction, re-exposure to high-intensity atorvastatin is not advisable. If lipid-lowering therapy is required, alternative statins not significantly metabolized by CYP3A4, such as rosuvastatin or pravastatin, may be considered to reduce the risk of recurrence [6,8]. More broadly, patients receiving high-dose CYP3A4-metabolized statins in combination with ticagrelor, particularly those with underlying renal impairment, may benefit from closer clinical and biochemical monitoring for early detection of muscle toxicity. This case demonstrates that significant toxicity can accumulate silently over weeks before becoming clinically apparent [2,4].

## Conclusions

This case highlights that severe statin-induced rhabdomyolysis can develop weeks to months after apparently well-tolerated high-intensity statin therapy, particularly when a CYP3A4 inhibitor such as ticagrelor is co-administered. Clinicians should remain vigilant beyond the early post-initiation period in high-risk patients. Although this drug interaction is uncommon, patients receiving high-dose atorvastatin and ticagrelor, particularly those with underlying renal impairment, may benefit from closer clinical and biochemical monitoring. Alternative statins with minimal CYP3A4 metabolism may be preferred, especially if co-administered with ticagrelor.

Early recognition, prompt discontinuation of the offending agent, aggressive intravenous hydration, and correction of metabolic abnormalities are essential and may prevent the need for dialysis. This case also emphasizes that severe non-traumatic rhabdomyolysis may be associated with progressive anemia and functional iron deficiency, which may necessitate iron supplements or blood transfusions.
